# Characterizing diamond detectors for various dose and dose rate measurements in scanned carbon and oxygen beams

**DOI:** 10.1002/mp.17893

**Published:** 2025-06-02

**Authors:** Celine Karle, Gianluca Verona‐Rinati, Stephan Brons, Rainer Cee, Stefan Scheloske, Christian Schömers, Rafael Kranzer, Thomas Haberer, Marco Marinelli, Andrea Mairani, Thomas Tessonnier

**Affiliations:** ^1^ Clinical Cooperation Unit Translational Radiation Oncology National Center for Tumor Diseases (NCT) Heidelberg University Hospital (UKHD) and German Cancer Research Center (DKFZ) Heidelberg Germany; ^2^ Department of Physics and Astronomy Heidelberg University Heidelberg Germany; ^3^ Industrial Engineering Department University of Rome “Tor Vergata” Rome Italy; ^4^ Heidelberg Ion‐Beam Therapy Center (HIT) Department of Radiation Oncology Heidelberg University Hospital Heidelberg Germany; ^5^ PTW‐Freiburg Freiburg Germany; ^6^ Department of Radiation Oncology Heidelberg University Hospital Heidelberg Institute of Radiation Oncology (HIRO) National Center for Tumor Diseases (NCT) University Hospital Heidelberg (UKHD) Heidelberg Germany; ^7^ National Center for Oncological Hadrontherapy (CNAO), Medical Physics Pavia Italy; ^8^ Clinical Cooperation Unit Radiation Oncology National Center for Tumor Diseases (NCT) Heidelberg University Hospital (UKHD) and German Cancer Research Center (DKFZ) Heidelberg Germany

**Keywords:** diamond detector, high LET, UHDR

## Abstract

**Background:**

The emerging FLASH radiotherapy technique employs “Ultra‐High Dose Rate” (UHDR) irradiations and offers the potential to spare normal tissue while maintaining iso‐effective tumor treatment. Given the physical and biological advantages inherent to high “Linear Energy Transfer” (LET) particles, the combination of UHDR and high LET has the capability to enhance the normal tissues sparing, as indicated by initial in vivo trials. However, to ensure a safe implementation of this combined modality, it is essential to establish robust dosimetric protocols utilizing dose‐, dose rate‐, and LET‐independent detectors.

**Purpose:**

The objective of this study is to characterize the dose, dose rate, and LET dependency of two diamond detectors with high LET carbon and oxygen ion irradiation under “Standard Dose Rate” (SDR) and UHDR conditions.

**Methods:**

The “microDiamond” (mD) and a “flashDiamond” (fD) prototype were benchmarked against measurements with a monitoring ionization chamber, Advanced Markus chamber (AMC), and simulations for carbon and oxygen irradiation, with energies of 274.98 MeV/u and 325.98 MeV/u under SDR and UHDR conditions. First, the entire depth‐dose profiles obtained during SDR irradiations and the partial in‐depth profiles of the Bragg peak region in UHDR were compared to the corresponding simulation values. Secondly, the linearity of the diamond detector response during dose escalation measurements was investigated for both dose rates.

**Results:**

The two detectors exhibited alignment with the simulated depth‐dose distributions for oxygen and carbon irradiations across both dose rate conditions. The mD overestimated the dose values for carbon and oxygen measurements. This overestimation increased with “dose‐averaged LET” (LETd) during SDR irradiation and maintained a stable value of 5% for UHDR. Meanwhile, the fD demonstrated a high degree of agreement with the simulation, with a maximum discrepancy of 5% across all irradiation modalities in the plateau and “Bragg Peak” (BP). Deviations were observed in the BP fall‐off region, while both diamond detectors exhibited a strong alignment with the AMC measurements. Furthermore, both detectors exhibited dose linearity under SDR and UHDR irradiation for both carbon and oxygen irradiation, with a coefficient of determination (*R* ^2^) above 0.99.

**Conclusion:**

In the context of heavy ion carbon and oxygen irradiation in UHDR and SDR, the two diamond detectors demonstrated dose‐rate independence. While the mD exhibited a tendency to overestimate dose values with increasing LETd, the fD was found to be LET‐independent. The fD appears to offer accurate and reliable dose assessments for UHDR heavy ion experiments.

## 1 INTRODUCTION

“Ultra‐high Dose Rate” (UHDR) irradiation offers a promising new avenue for treatment modalities. As early as the 1960s, irradiation with dose‐rates exceeding 40 Gy/s, classified as UHDR, have been found to spare cells to a greater extent than “Standard Dose Rate” (SDR) irradiation when the same dose is applied.^1^ The general cell sparing ability of UHDR irradiation has been the subject of extensive investigation for electrons and light ion, such as protons^2^ and helium ions.^3^ Furthermore, in vivo irradiation at UHDR reduces normal tissue toxicity while maintaining effective tumor control, which is termed as “FLASH effect”.^4^, ^5^ Promising in vitro and in vivo results led to the first clinical trials with patients receiving electron^6^ and proton^7^ UHDR irradiation.

In comparison to light ions, heavier particles like carbon ions, exhibit both physical advantages in providing more precise dose profiles, as well as biological advantages due to their increased “Linear Energy Transfer” (LET) in the “Bragg Peak” (BP) region. The higher LET aids in eradicating radioresistant and hypoxic tumors, which makes the use of even higher mass particles, such as oxygen, attractive.^8^, ^9^, ^10^

Combining UHDR treatment with heavy ions could allow to take advantage of both approaches.^11^, ^12^ A recent study by Tinganelli et al. showed promising results for carbon UHDR irradiations in both low LET entrance^13^ and high LET BP region.^14^

While, the precise mechanism responsible for this phenomenon remains unclear, the temporal structure of the dose delivery might play an important role in the FLASH effect,^15^, ^16^ for instance as demonstrated by Ruan et al., who examined the impact of altering the beam pause between two pulses and the mean dose rate on crypt survival.^16^ Similarly, Karsch et al. investigated the impact of different temporal settings on zebra fish embryo lengths.^17^ These are merely two illustrations of the significance of precise temporal recording to enhance understanding of the FLASH effect. Concerning the temporal beam delivery, the beam time structure of the diverse FLASH delivery systems, such as isochronous cyclotrons and synchrotrons, differs^18^ and especially for inter‐institutional comparison the logging of all the parameters, like spill time, interspill pauses, and pulse frequencies is crucial.^19^ Temporal monitoring is of particular interest in the context of scanned ion beams, where instantaneous dose rates can reach exceptionally high levels.^20^, ^21^ Consequently, the analysis of spatio‐temporal dose distribution is crucial and demonstrates the necessity of suitable detectors that can provide time‐resolved measurements while remaining independent of dose, dose rate, and LET.

Passive dosimetric systems, such as alanine detectors, thermoluminescence detectors, or radiochromic films, may be suitable for the assessment of absolute dose; however, they do not permit active temporal monitoring of the dose during the course of the experiments.^22^, ^23^ This can only be evaluated through the use of active real‐time systems with a small time resolution.

The conjunction of UHDR and high LET necessitates the implementation of dosimetric systems that circumvent the physical demands of these radiation modalities, such as saturation and recombination effects.^12^ In the case of high LET, the resulting recombination charge loss can be attributed to two principal factors: volume recombination, where ions from different primary particles recombine, and initial recombination, where ions produced by the same primary particle interact.^24^ Although the volume recombination appears to be insignificant in the context of high LET irradiation and heavy ions, the intra‐track effects are amplified.^12^, ^24^

“Ionization Chambers” (IC) are recommended by the IAEA as a reference for high LET irradiation dosimetry.^25^ However, ICs are susceptible to saturation in the context of rapid charge releases following UHDR irradiation, due to the influence of recombination^22^, ^26^, ^27^ and polarization effects.^28^ Despite the existence of methodologies for modelling ion recombination within an IC, this approach is not applicable to synchrotron beams, due to their non‐reproducible intensity fluctuations and heterogeneous high instantaneous dose rates.^18^ To circumvent the application of numerous correction factors,^22^, ^29^ hardware adaptations have been investigated, including the use of alternative gas mixtures in the chamber^30^ or the reduction of the gap between the electrodes.^31^ Despite these promising advancements in signal acquisition, the response time of available ICs is around 300 µs, which is too slow to resolve the single‐digit microsecond beam time structure of certain isochronous and synchrotron delivery systems.^18^ Furthermore, the low spatial resolution due to significant volume averaging effects poses major challenges for UHDR applications, which often involve small fields with large dose gradients.^23^

A preferable higher resolution and thus smaller sensitive volumes can be achieved with more dense solid‐state detectors.^32^ High spatial resolution in the nano‐ to micrometer range can thus be attained.^18^ In the context of solid state detector materials, scintillators are inherently constrained by their dead times due to the nature of their scintillation centers and optical contamination by Cherenkov light in the optical fibers.^23^ These factors could result in unintended LET and dose rate‐dependent effects.^22^, ^27^ Additionally, high LET irradiation has been observed to cause ionization quenching in scintillators, necessitating additional corrections to be made.^33^, ^34^, ^35^ Furthermore, scintillators can experience radiation damage, leading to a decrease in light output and a shift in spectra.^36^

Regarding radiation hardness, semiconductors are highly resistant to radiation damage and additionally offer the advantage of a direct charged based read out that should not be less affected by high LET and dead times.^22^, ^23^ Their small active volume and resulting high axial resolution allows for the assessment of detailed lateral and “Depth Dose Distributions” (DDD).^37^ Thus, semiconductors have a high sensitivity due to the charge‐based signal, a high spatial resolution, and thus are suitable for monitoring the temporal application of UHDR small fields.^18^, ^23^

Silicon (Si) diodes represent a class of semiconductor detectors that are currently available. However, the high‐Z material results in considerable variation of mass energy absorption coefficient and electronic stopping power when compared to tissue equivalent materials. This leads to an energy‐dependent outcome and introduces general uncertainties.^38^ Radiation damage can also be expected for Si diodes under UHDR conditions, especially in combination with high LET, albeit to a lesser extent than scintillators.^39^

These disadvantages are avoided when diamond is selected as a sensitive component for the semiconductor detector. The diamond crystal is near tissue equivalent and resistant to radiation damage.^23^, ^40^ Diamonds as semiconductor material exhibit a wider band gap. Consequently, this results in reduced leakage currents and an enhanced charge collection efficiency compared to silicon‐based semiconductors.^41^ Currently available diamond‐based detectors are the “microDiamond” (mD)^42^ and “flashDiamond” (fD) both from PTW.^43^

Recent advancements in the field have seen the development of silicon carbide (SiC) detectors, which combine the industrial maturity of silicon and the robustness of diamonds. Initial studies have demonstrated the potential of SiC detectors in the context of electron UHDR irradiation, yielding results that are comparable to those observed in fD.^44^ However, further research is required in the characterization of SiC detectors under high LET irradiation.

The commercially available mD from PTW^42^ has already been tested and found to be dose rate independent for photon, electron, and helium irradiation.^45^, ^46^ Furthermore, the mD was benchmarked for scanned carbon ions during SDR irradiation, confirming a negligible LET dependence for this range.^37^ However, in the course of investigating unmodulated beams, Rossomme et al.^47^ found an LET dependency of low‐energy carbon and oxygen ions with 62 Mev/u. This discrepancy could be attributed to the presence of a border LET spectrum in beams with modulators, such as a ripple filter, beam monitoring systems, and so forth.^47^

Due to its promising characteristics for UHDR real‐time beam monitoring, the mD was therefore tested in this study. Given indications that the mD might be affected by high LET and UHDR irradiaton,^48^ additionally, a prototype of a “fD” from PTW was investigated. The fD uses the base of an mD, but was developed to fulfill the UHDR requirements with modifications made to the sensitive area and the diamond‐doped area.^49^ In order to guarantee that the diamond Schottky diode provides a linear response at high dose per pulse without saturating, two approaches were employed: the first is to limit the detection current, thereby reducing sensitivity; the second is to decrease the series resistance. The detection current can be reduced by decreasing the sensitive area, while the series resistance is minimized by increasing the boron concentration in the doped diamond layer.^49^ These modifications introduced have enabled the fD to record a reliable signal under electron and helium UHDR irradiation^45^, ^46^ and thus showcased its potential to serve potentially as a secondary standard instrument for absolute dose measurements in water for UHDR beams.^48^

The objective of this study was to investigate whether these UHDR adaptations are also appropriate for heavy particle UHDR irradiation. Accordingly, the mD and fD were analyzed regarding their dose linearity and depth dose response under SDR and UHDR irradiation with carbon and oxygen ions at the “Heidelberg Ion beam Therapy facility” (HIT) to evaluate the detectors’ reliability for combined high LET and high dose rate irradiation.

## 2 MATERIALS AND METHODS

### 2.1 Detectors and electronic chains

Two diamond detectors were tested using different dose rates and LET: the commercially available “mD” (TM60019, PTW Freiburg)^42^ and a “fD” prototype, while the latter has been optimized for UHDR applications. Both detectors contain an active volume comprising a pure intrinsic diamond layer mounted on top of a conductive p‐type boron doped diamond layer.^49^, ^50^ The detectors differ mainly in their boron concentration in the p‐type layer and the size of the active volume.^49^ The mD has an active layer with a diameter of 2.2 mm and a thickness of 1 µm,^37^ while the fD prototype has a similar geometric structure but a 1.5 smaller active area (diameter 1.4 mm).^45^ Both detectors have a similar construction and are encapsulated, resulting in a measurement point situated at a water equivalent depth of 1 mm. Due to their intrinsic build‐up potential, no external voltage needs to be applied.^37^, ^49^ The response signal of the mD remained stable when rotated around the azimuthal axis. However, it was necessary to maintain the detectors' polar angle at 0° in order to circumvent any potential uncertainties.^40^

The mD demonstrated reliable signal generation under conditions of SDR high‐LET irradiation,^37^ as well as for UHDR eletrons,^45^ proton^26^ and helium^46^ irradiation. Under these conditions, the signal linearity, as specified by PTW, was achieved. Similarly, the fD prototype was validated for UHDR irradiation with electrons and helium ions.^45^, ^46^

In accordance with the recommendations set forth in TRS‐398, SDR measurements were additionally conducted with a plan‐parallel “Advanced Marcus Chamber” (TM34045, PTW Freiburg) (AMC) in axial orientation.^25^ The AMC has a sensitive volume with a radius of 2.5 mm and a depth of 1 mm. To compare the values of the three detectors, “Monte Carlo” (MC) FLUKA (Version 2021.2.9) simulations were conducted to obtain DDD and LET distributions in a water tank using a detailed geometry of the HIT beamline.^51^, ^52^

For the electronic readout, prior studies demonstrated that the diamond detectors offer a reliable readout during SDR and UHDR irradiation when coupled with the PTW UNIDOSE^webline^ electrometer.^37^, ^45^, ^46^ The charge was recorded for the diamond detectors, whereas for the AMC, the dose was calculated directly using the calibration factor of the chamber and the correction factor for radiation quality with an applied temperature and pressure correction.

### 2.2 Experiments and evaluation

Two key experiments were carried out to assess the dose, dose rate, and LET dependencies of the two diamond detectors. The first experiment involved measuring the entire DDD under SDR irradiation and focusing on the BP region under UHDR irradiation. The second experiment evaluated the linearity of the dose response during dose escalation measurements for the diamond detectors in both SDR and UHDR settings.

### 2.3 Setup

**FIGURE 1:**
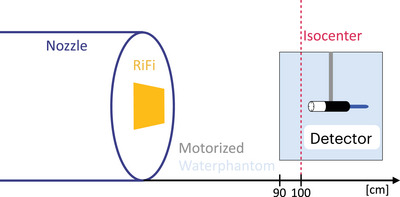
Setup at the HIT experimental room: The beam exit nozzle is equipped with a 3 mm ripple filter (“RiFi”). A motorized water phantom is positioned with its entrance window 10 cm before the isocenter, marked with a red line. All three detectors are positioned with dedicated holders into the moving metal arms of the water phantom.

The three different detectors were positioned with their designated holders inside a motorized water tank. The phantom was positioned at approximately 10 cm in front of the iso center at the “Heidelberg Ion Beam Therapy Center” (HIT) experimental room (Figure 1).

### 2.4 Depth dose distribution

**FIGURE 2:**
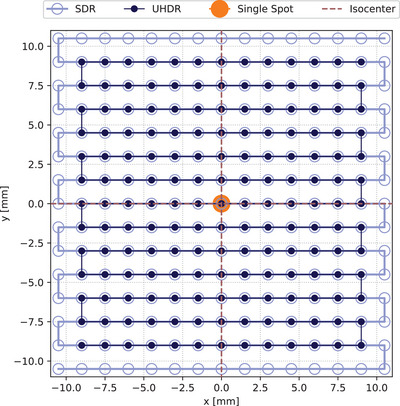
“PBS” patterns of the fields in beam's eye view. All fields for carbon and oxygen irradiations were centered in the isocenter, indicated by the dashed red lines, and had a spot spacing distance of 1.5 mm. The PBS direction is depicted by solid lines. For all SDR experiments, the 21 × 21 cm^2^ field was used (marked as light blue). The fields employed for the UHDR DDD measurements were of a slightly reduced size in comparison to those utilized in SDR, with the objective of increasing the dose rates (dark blue). The single spot plans, displayed as orange spot, were used for the dose escalation experiment with UHDR. The varying spot sizes have been selected for enhanced visualization. DDD, depth dose distributions; PBS, pencil beam scanning; SDR, standard dose rate; UHDR, Ultra‐High Dose Rate.

The delivered dose or charge was measured from irradiated fields (Figure 2 and Table 1) along the central axis 4 cm up to ∼ 17 cm in water depth with all three detectors in SDR. The BP region was the focus of the UHDR measurements. For each depth point, three measurements were averaged, and the standard deviation was calculated. The dose or charge values were normalized to the “Area Under the Curve” (AUC). The AUC was calculated by interpolating the measured values and then integrated using the trapezoidal rule to obtain the total AUC. For the UHDR measurements, where only the Bragg peak was acquired, the values from these measurements were normalized to those from the SDR measurements and subsequently divided by the SDR AUC value. The standard deviations were determined through Gaussian error propagation. Subsequently, the local deviation from the MC values was calculated with a symmetric percentage change formular.

**TABLE 1:** The various experimental settings.

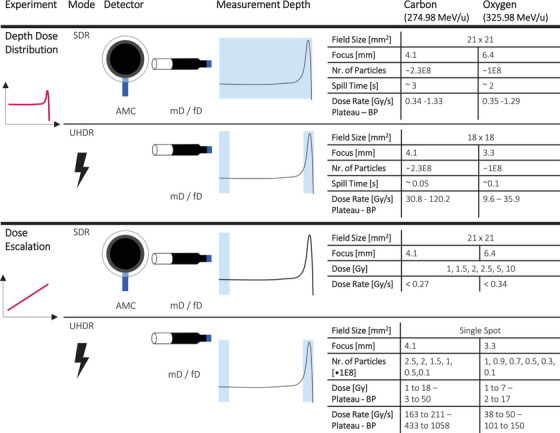

*Note*: The blue area in the measurement depth column indicates the location of the detectors. The plan details include field size and focus, expressed as the full width at half maximum. Depending on the experiment, the number of particles applied, or the dose is provided. The dose rate given represents the mean dose rate, which was calculated by dividing the total dose applied by the irradiation time. For the dose escalation for SDR the highest and for UHDR the lowest reached dose rate is provided.

### 2.5 Dose response linearity

To determine the linearity of the SDR dose response for carbon and oxygen, field plans (Figure 2) were calibrated to deliver doses of 1, 1.5, 2, 2.5, 5, and 10 Gy using the AMC (Table 1) in the plateau region. During the calibration process, the AMC doses were recorded a total of three times. Similarly, the charge from the diamond detectors was recorded three times for each dose in each plan, with the resulting values averaged. The charges and their standard deviations were then fitted linearly using an orthogonal distance regression accounting for the x‐deviation provided by the AMC dose measurement.

The slope of this fit represents a sensitivity factor that correlates the dose obtained by the AMC to the charges from the diamond detectors. The factors for carbon and oxygen were then applied to convert the charges for the different UHDR experiments into dose, allowing the calculation of the dose and dose rate.

In order to achieve high doses and dose rates at UHDR, a single spot on the central axis was irradiated in opposition to the conventional approach of using a large field (Figure 2). Given the relatively small field size, the actual number of particles delivered was used as the reference for linearity rather than the AMC. A single spot with the desired quantity of particles was delivered for carbon with approximately 2.5 × 10^8^, 2 × 10^8^, 1.5 × 10^8^, 1 × 10^8^, 0.5 × 10^8^ and 0.1 × 10^8^ particles and for oxygen with approximately 1 × 10^8^, 0.9 × 10^8^, 0.7 × 10^8^, 0.5 × 10^8^, 0.3 × 10^8^ and 0.1 × 10^8^ particles. The “Beam Application and Monitoring System” (BAMS) system at HIT recorded the actual number of particles delivered, which differed from the requested amount due to the instability of the synchrotron settings. Consequently, the charge was normalized to the actual number of particles delivered. As the variation in particle delivery was already accounted for, the data was fitted by a linear function using the least squares method.

### 2.6 Dose rate calculation

In the case of the SDR irradiations, the dose values were provided by the AMC. Conversely, for the UHDR experiments, the sensitivity factor was employed to convert the charges to dose values. The irradiation times were determined using the recorded BAMS files, and the average dose rate was calculated by dividing the dose by the total irradiation time.^19^ The reported dose rate values are based on at least three different BAMS recordings and corresponding dose measurements (Table 1).

### 2.7 Film analysis

**FIGURE 3:**
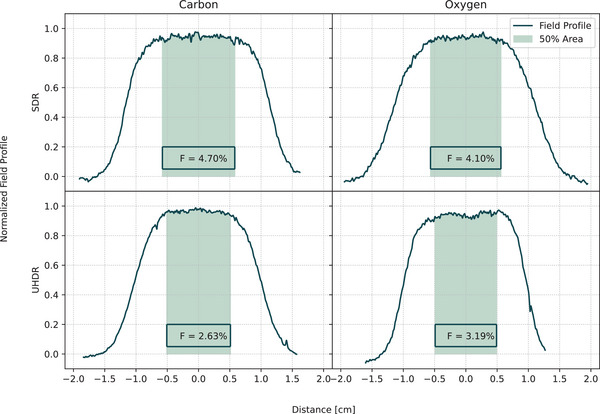
Normalized field profiles of the EBT3 films. The evaluated 50% area is displayed as green shaded region, in which also the flatness index is given.

Given that the irradiation was conducted with an active “Pencil Beam Scanning” (PBS) system, it is essential to ascertain the level of flatness exhibited by the field in the region of the detectors. Therefore, EBT3 Gafchromic Films (Ashland) were placed in front of the water tank during irradiation. Each film was scanned with an Epson Perfection V850 Pro at a resolution of 1200 dpi. Horizontal and vertical line profiles through the center of the film were extracted with ImageJ. The resulting data were then subjected to further processing in Python, whereby the profile was normalized to the maximum greyscale value and smoothed with a Gaussian filter. From the central point of the profile, we identified a region spanning 50% of the total area under the curve (highlighted in green in Figure 3) to exceeds the dimensions of the sensitive areas of all detectors. Within this central region, we calculated a flatness index (F):

$$F=100*\frac{Y_{\max}-Y_{\min}}{Y_{\max}+Y_{\min}}$$

with *Y*max and *Y*min being the maximum and minimum normalized grey scale value in the 50% area.

## 3 IRRADIATION DETAILS

### 3.1 Irradiation facility

The HIT provides intensity‐controlled raster scanning pencil beams consisting of proton, helium, carbon, and oxygen ions with energies between 50 and 430 MeV/u.^53^ A third‐order resonant RF knock‐out extraction system is responsible for the selective extraction of the requested number of particles from the synchrotron. The extracted particles are then transported to the dedicated room, for this study the experimental room. During the extraction phase, the dynamic intensity controller modulates the amplitude of the RF‐knockout exciter in response to detected deviations.^54^ To extract carbon and oxygen ions with UHDR at HIT, it was necessary to implement a series of adaptations with the objective of increasing the number of particles and reducing the extraction time. Consequently, the synchrotron was filled with a certain number of particles that can be extracted in one spill, which was 2.5 × 10^8^ and 1 × 10^8^ for carbon and oxygen ions respectively. The extraction tune was adjusted to a position closer to the resonance, and the field strength of the sextuple magnets was enhanced to facilitate a more rapid extraction. These modifications did not affect the operational status of the PBS and intensity control systems, which remained functional for UHDR irradiation.^55^

Additionally, the nozzle was equipped with a 3 mm ripple filter to broaden the pristine BP for both carbon and oxygen beams and with a BAMS containing several IC and multi‐wire chambers (Figure 1). These chambers are employed for the control of beam delivery,^54^ the assessment of the spill length, and the number of particles that were delivered.

### 3.2 Irradiation parameters

The HIT accelerator provided carbon ions with an energy of 274.98 MeV/u or oxygen ions with 325.98 MeV/u for both SDR and UHDR irradiations. For the beam application at HIT, active PBS is used, and the spot spacing between the pencil beams was 1.5 mm for both particles and dose rates. The various experiment settings and irradiation parameters for the DDD and dose escalation experiments at SDR and UHDR are presented in Table 1.

## 4 RESULTS AND DISCUSSION

### 4.1 Field homogeneity

The field homogeneity was evaluated by analyzing irradiated EBT3 films. The extracted profile lines and the central 50% area are shown in Figure 3. Despite the active PBS application of the fields, the small flatness factor below 5% indicates that the irradiation in the area considered was homogeneous. As this area extended beyond the sensitive area of the detectors, the detectors were adequately covered by both SDR and UHDR fields.

### 4.2 Depth dose response

Depth dose profiles were recorded in SDR for the AMC, mD, and fD, and in UHDR for the diamond detectors in both the plateau and BP regions. Dose rates for carbon UHDR irradiation ranged from 30.8 to 120.2 Gy/s in the plateau and the BP respectively, while for oxygen UHDR, even in the BP region the dose rate did not exceed 35.9 Gy/s (Table 1). It is important to note that the average dose rate is not the sole indicator of dose rate, particularly for PBS systems.^19^, ^46^ Due to the raster scanning process, only a few surrounding spots contribute to the local dose, meaning that the actual irradiation time locally may be much shorter than the total field irradiation time. This effect has been previously demonstrated for helium,^46^ highlighting the need for further time‐resolved studies to accurately determine the instantaneous dose rate pattern and other indicators.^21^

**FIGURE 4:**
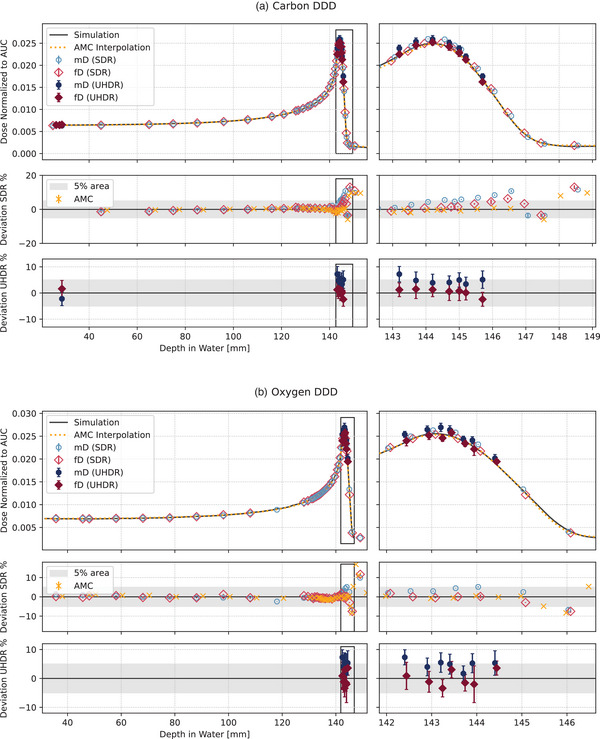
Carbon and oxygen DDD normalized to the AUC. The upper plot A displays the carbon data, while the lower panel B contains the data from oxygen irradiations. For each individual panel, the left side displays the full DDD, and the right side specifically focuses on the BP and BP fall of region. The first plot depicts the dose values normalized to the AUC, the middle plot illustrates the percentage local deviation from SDR measurements in relation to FLUKA simulation, shown as back lines, and the bottom graph represents the deviations from UHDR diamond measurements in comparison to the simulations. The AMC values are shown as dotted orange line resulting from a quadratic interpolation of the individual values for better visibility in the DDD plots, and for the deviation plots, the individual measurement points are marked with orange crosses. The data points for the mD (blue circle) and fD (red diamond) are shown individually. The UHDR measurements are marked by a darker color and a filled marker symbol. Additionally, the 5% deviation area is marked with a grey band. AUC, Area Under the Curve; BP, Bragg Peak; DDD, depth dose distributions; fD, flashDiamond; mD, microDiamond; UHDR, Ultra‐High Dose Rate.

The recorded DDD were normalized to the AUC and are displayed in Figure 4. In addition to the individual deviations shown in Figure 4, the average deviations from the simulations in the BP region for all detectors at SDR and UHDR are given in Table 2.

**TABLE 2:** Average percentage deviation in the BP region from the FLUKA simulation: Values are given with their standard deviation.

Particle	Dose Rate	Detector	Average Deviation ± Standard deviation [%]
Carbon	SDR	AMC	0.81 ± 0.08
		mD	4.53 ± 0.29
		fD	2.5 ± 0.4
	UHDR	mD	3.9 ± 2.2
		fD	0.6 ± 2.2
Oxygen	SDR	AMC	−0.08 ± 0.15
		mD	2.1 ± 0.3
		fD	−1.0 ± 0.4
	UHDR	mD	3.4 ± 2.4
		fD	−1.0 ± 2.6

Abbreviations: BP, Bragg Peak; fD, flashDiamond; mD, microDiamond; SDR, standard dose rate; UHDR, ultra‐high dose rate.

In general, while the standard deviations for SDR measurements for all detectors were below 1%, the standard deviations of UHDR measurements were around 3%, due to the variability of the delivery (Figure 4). This can also be seen by the variability of the average deviation, which was for SDR below 0.2% and for UHDR around 2.5% (Table 2). The adjustment of the synchrotron to deliver UHDR results in the particles being moved closer to the extraction tune. This adaptation can lead to more variable dose delivery compared to the normal particle delivery.

It is noteworthy that the average deviation of all detectors in the BP region remained below 5% (see Table 2), indicating a high degree of agreement with the simulations, even at this high LET and UHDR. The observed consistency in the average deviation of the diamonds between SDR and UHDR (see Table 2) suggested a dose rate independence for the conducted high LET carbon and oxygen irradiations.

The AMC dose values corresponded to the simulation values and resulted in individual deviations below 2%, except for a few measurement values at the distal part of the BP. Several factors may be responsible for this partial discrepancy. First, the nuclear model of FLUKA may have limitations in reproducing the depth dose distribution, which is of particular relevance for carbon and oxygen beams, due to a lack of experimental fragmentation data.^56^, ^57^ Second, the AMC dose measurements were affected by the experimental conditions with dose variations in the order of 0.4%, but in general, uncertainties in the dosimetry of heavy ions may reach 3.4% in the absorbed dose in water due to uncertainties in the stopping power ratio.^25^

**FIGURE 5:**
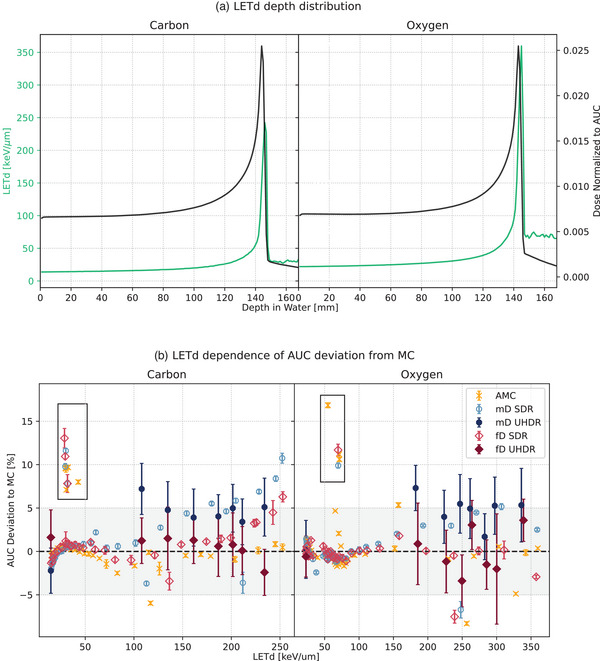
LETd dependency of the AUC deviation with respect to the MC. The data related to carbon is displayed on the left‐hand side of the panels, while the data pertaining to oxygen is displayed on the right‐hand side. Panel A depicts the LETd distribution (green solid line) alongside the AUC distribution (black solid line). Panel B illustrates the resulting correlation between AUC deviation and MC, as well as LETd at varying depth values. As previously, AMC values are represented by orange crosses, mD by blue circles, and fD by red diamonds. Hollow markers indicate SDR irradiation data, while filled markers represent UHDR measurement points. The black boxes surround the values from the fragmentation tail. AUC, Area Under the Curve; AMC, Advanced Markus chamber; LETd, dose‐averaged LET; MC, Monte Carlo; mD, microDiamond; SDR, Standard Dose Rate.

The mD displayed around 4% overestimation of the dose compared to the simulation in the BP region for both SDR and UHDR and both particles (Table 2). This is consistent with the findings found by Tessonnier et al^46^ for helium irradiation. As shown in Figure 5b, the deviation to the simulation for carbon and oxygen irradiations tended to increase for SDR irradiations with “dose‐averaged LET” (LETd), indicating an LETd dependency. However, the largest variations up to 15% for carbon and 12% for oxygen (Figure 5b, black box) were at an LETd of about 30 keV/µm and 60 keV/µm for carbon and oxygen respectively, which corresponded to the tail of the DDD (Figure 5a). This region is highly influenced by the nuclear model used in FLUKA, as evidenced by the fact that all detectors show similar deviations from the simulation in this area. The consistency between the AMC, mD, and fD detectors further supported this observation.

For the UHDR irradiations with both ions, deviations of the mD were around +5% for all LETd within the BP region, without a possibility to observe the trends shown in SDR due to the larger uncertainty of the measurements (Figure 5b). When considering the consistency of the average deviation of the mD for SDR and UHDR for both particle types (Table 2), this suggested that the mD may have an LETd but no dose rate dependency.

The deviations of the fD for carbon and oxygen in UHDR and SDR were below 5% until the fall‐off of the BP. For carbon ions, the dose was generally slightly overestimated, though the deviations were less than half of those seen for the mD values. This overestimation wasnot observed in the oxygen DDD. As shown in Figure 5, for oxygen no clear LETd ​trend was apparent for both SDR and UHDR, indicating that the detector was independent of LETd​ and dose rate. Moreover, the average deviation for the fD in the BP region was 2.5% at most, which was generally smaller than the mD deviations (Table 2). Furthermore, it is noteworthy that the discrepancies from the simulation were consistent between UHDR and SDR, indicating a dose rate independence.

**FIGURE 6:**
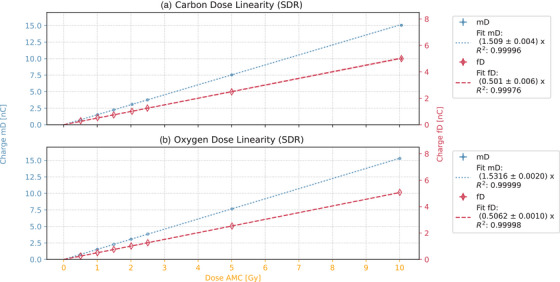
Dose linearity for SDR field irradiation for carbon in the upper plot and for oxygen in the lower plot. The data points for mD and fD are presented with their respective standard deviations. The right‐hand side legends provide the fit parameter value, its standard deviation, and the *R* ^2^ for the fit. The 1σ standard deviation is included in the plots as a shaded area. mD, microDiamond; fD, flashDiamond.

### 4.3 Dose response linearity SDR

The detector charge was benchmarked against the AMC dose values of the irradiated PBS fields to evaluate the linearity of the detector response in the SDR irradiations within the plateau region (Figure 6). The detectors demonstrated an overall dose linearity, as indicated by coefficients of determination (R^2^) of the linear fit exceeding 0.99. The mD demonstrated a sensitivity of 1.509^nC^/_Gy_ for carbon and a marginal increase in sensitivity for oxygen, reaching 1.5316^nC^/_Gy_. This value was threefold higher than those observed for the fD, which yielded 0.501^nC^/_Gy_ for both particle types within the standard deviation. Therefore, neither of the diamond detectors exhibited dose dependence during SDR irradiation.

### 4.4 Dose linearity UHDR

**FIGURE 7:**
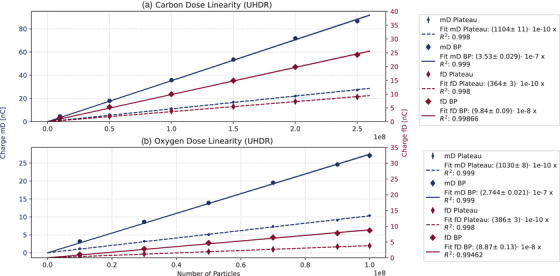
Dose linearity after UHDR single spot irradiation for carbon in the upper plot and for oxygen in the lower plot. The data points for mD and fD are presented with their respective standard deviations. The measured values for the plateau are marked with smaller symbols and a dashed line, while for the BP measurement a with larger symbols and a solid line was chosen. The right‐hand side legends provide the fit parameter value, its standard deviation, and the *R* ^2^ for the fit. The 1σ standard deviation is included in the plots as a shaded area. BP, Bragg Peak; mD, microDiamond; UHDR, Ultra‐High Dose Rate.

For the UHDR dose response linearity, the detector response was compared to the delivered particles applied to a single spot on the central axis (Figure 7).

Similarly to the SDR results, the diamonds demonstrated satisfactory linearity during UHDR dose escalation measurement within both plateau and BP region. Particularly the coefficient of determination for the various linear fits exceeded 0.99.

The elevated UHDR standard deviations in comparison to the SDR measurements can be explained by the beam fluctuations, due to UHDR delivery conditions.

## 5 CONCLUSION

The two diamond detectors exhibited reliable and accurate dose measurements during heavy ion irradiations, thereby confirming their strong potential for use in UHDR dosimetry. While the mD showed an increasing deviation with higher LETd, the fD prototype demonstrated superior accuracy and an absence of dose, dose rate, and LET dependency, making it an appropriate choice for the assessment of the absolute dose in high‐LET irradiations at UHDR. In comparison with other detector candidates that might be suitable for these radiation modalities, such as SiC detectors, the fD is commercially available and can be integrated into normal dose‐metric routines by being connected to standard electrometers.^44^, ^45^, ^58^ Consequently, the fD is readily available for research in this area and might be suitable as a secondary standard.^48^

A fundamental concern in the investigation of the FLASH effect and its clinical applicability is the deciphering the temporal effects of beam application. Consequently, reliable real‐time beam monitoring with devices such as the fD is becoming increasingly important.^20^ This capability allows precise instantaneous dose rate measurements when coupled to a digital oscilloscope, which is crucial for investigating the role of dose delivery structure in the FLASH effect during heavy ion therapy.

## CONFLICT OF INTEREST STATEMENT

Rafael Kranzer is a PTW employee, while Marco Marinelli and Gianluca Verona‐Rinati signed a contract with PTW‐Freiburg involving financial interests derived from the PTW diamond detector's commercialization. The funding sources had no role in the study design, data collection, analysis, interpretation, or the decision to submit the results for publication. All other authors declare that there are no conflicts of interest regarding the publication of this paper.

## Data Availability

Research data is stored in an institutional repository and will be shared upon request to the corresponding author.
